# Fate of Sulfonamides and Tetracyclines in Meat during Pan Cooking: Focus on the Thermodegradation of Sulfamethoxazole

**DOI:** 10.3390/molecules27196233

**Published:** 2022-09-22

**Authors:** Christelle Planche, Sylvie Chevolleau, Maria-Hélèna Noguer-Meireles, Isabelle Jouanin, Sophie Mompelat, Jérémy Ratel, Eric Verdon, Erwan Engel, Laurent Debrauwer

**Affiliations:** 1 INRAE, UR370 QuaPA, MASS Group, F-63122 Saint-Genès-Champanelle, France; 2 Toxalim, Université de Toulouse, INRAE UMR 1331, INP-ENVT, INP-EI-Purpan, UPS, F-31027 Toulouse, France; 3 Axiom Platform, MetaboHUB-MetaToul, National Infrastructure of Metabolomics and Fluxomics, F-31027 Toulouse, France; 4 ANSES, French Agency for Food, Environmental and Occupational Health & Safety, Laboratory of Fougères, Javené, F-35306 Fougères, France

**Keywords:** sulfonamides, tetracyclines, radiolabeling, degradation products, cooking, beef meat

## Abstract

Although antimicrobials are generally found in trace amounts in meat, the human health risk they bear cannot be ignored. With the ultimate aim of making a better assessment of consumer exposure, this study explored the effects of pan cooking on sulfonamides and tetracyclines in meat. Screening of these antimicrobials in cooked meat was first performed by the European Union Reference Laboratory on the basis of HPLC-MS/MS analyses. A proof of concept approach using radiolabeling was then carried out on the most cooking-sensitive antimicrobial—sulfamethoxazole—to assess if a thermal degradation could explain the observed cooking losses. Degradation products were detected thanks to separation by HPLC and monitoring by online radioactivity detection. HPLC-Orbitrap HRMS analyses completed by 1D and 2D NMR experiments allowed the structural characterization of these degradation compounds. This study revealed that cooking could induce significant antimicrobial losses of up to 45% for sulfamethoxazole. Six potential degradation products of ^14^C-sulfamethoxazole were detected in cooked meat, and a thermal degradation pattern was proposed. This study highlights the importance of considering the cooking step in chemical risk assessment procedures and its impact on the level of chemical contaminants in meat and on the formation of potentially toxic breakdown compounds.

## 1. Introduction

Veterinary drugs are extensively used in food animal production for therapeutic and prophylactic purposes [1,2]. Among these drugs, sulfonamides and tetracyclines are two of the most commonly used antimicrobials [3]. These compounds are broad-spectrum antimicrobials widely used for the prevention and treatment of food-producing animals to cure several infectious diseases or used as feed additives to promote animal growth [4,5,6]. For each antimicrobial, the dosage and the recommended withdrawal period depend on the veterinary drug used and on the target species. However, residues of these compounds may remain in the animal food products if these drugs are incorrectly used or if recommended drug withdrawal periods are not observed [7,8,9]. The Commission Regulation (EU) N° 37/2010 of 22 December 2009 sets the maximum residue limits (MRLs) of veterinary drugs in foodstuffs of animal origin of 100 µg.kg^−1^ of meat for sulfonamides and tetracyclines [10]. Although antimicrobials are generally found in trace amounts in animal-derived food, the human health risk they bear cannot be ignored [11,12]. Currently, this risk is most often assessed from their concentration levels in raw foods. However, such knowledge alone does not enable a precise assessment of the consumers’ exposure because levels of antimicrobials can be affected by the thermal processing the food undergoes before consumption [13,14,15].

A wide examination of literature data regarding the effects of cooking on the degradation of sulfonamides and tetracyclines shows major discrepancies between reported results [16,17,18,19,20,21,22,23]. For instance, Ibrahim et al. [16] reported an approximate 80% decrease in oxytetracycline in ground lamb after 20 min boiling, whereas Abou-Raya et al. [19] found that 20 min boiling induced 51.5% loss of oxytetracycline in thigh meat. For sulfadiazine, Furusawa et al. [17] found no significant reduction in this anti-infective in chicken muscles after 12 min roasting, whereas Ismail-Fitry et al. [18] reported a 37.5% loss after deep-frying chicken meatballs at 190 °C for 9 min. At least two factors may explain these discrepancies. First, there are no standardized cooking protocols, and cooking conditions could significantly vary between different studies. The extent of these variations is difficult to assess because cooking protocols are generally poorly documented, with little or no information regarding temperature monitoring and control. Second, most of these studies deal with nonhomogeneous naturally contaminated food matrices which are not uniformly contaminated. Because of these discrepancies, no clear conclusion can be drawn regarding the effect of cooking on antimicrobials. Thus, these former studies are not complete and reliable enough to feed the risk assessment models, which require highly robust, comparable, and reproducible data. To address this challenging issue, a realistic, standardized, and reproducible cooking method must be set up [24]. It is also necessary to use a homogeneous matrix intentionally and uniformly contaminated at a known and high enough concentration to be detected, even after cooking. For sulfonamides and tetracyclines, little is known about their thermal degradation; it is, therefore, essential to determine their potential toxic breakdown compounds and to consider their presence in cooked meat when maximum residue limits are established [25].

With the ultimate aim of obtaining a better assessment of risks related to chemical contamination of food, we have studied the impact of cooking on sulfonamides and tetracyclines in meat. On the basis of HPLC-MS/MS analyses performed by the European Union Reference Laboratory, the first part of this paper is focused on the impact of a realistic “medium” cooking on the concentration of 15 sulfonamides and 6 tetracyclines in contaminant-spiked meat. The validity of these results obtained with spiked meat is discussed in light of contaminant levels measured after cooking in naturally incurred meat samples. To go further, a proof of concept approach using radiolabeling was proposed and carried out on the most cooking-sensitive antimicrobial (sulfamethoxazole) to assess if a thermal degradation could explain, at least in part, the observed antimicrobial cooking losses. The fate of ^14^C-sulfamethoxazole during cooking was investigated thanks to separation by HPLC and monitoring by online radioactivity detection. HPLC-Orbitrap HRMS analyses were completed by 1D and 2D NMR experiments, then allowed the structural characterization of sulfamethoxazole breakdown products, and a thermal degradation pattern was proposed.

## 2. Results and Discussion

### 2.1. Assessment of the Loss of Sulfonamide and Tetracycline Residues during Meat Cooking

To determine the impact of domestic cooking on sulfonamides and tetracyclines, the levels of these antimicrobials were determined by HPLC-MS/MS in spiked ground beef before and after a “medium” pan cooking (core 70 °C). Table 1 and Table S1 (Supplementary Material) display the concentrations obtained (in µg.kg^−1^ of meat, classical expression of veterinary drug concentration in food for risk assessment) and the corresponding antimicrobial cooking losses [26] and processing factors [27]. These tables show that antimicrobials display three different behaviors according to their family and structure. First, a significant increase in antimicrobial concentration during cooking (*p* < 0.05) was observed for 12 sulfonamides and 2 tetracyclines with processing factors ≥1.3. For these compounds, the mean weight loss of ground beef patties during cooking (34.0 ± 1.5% considering the mass of meat (g) before and after medium cooking in the present study) exceeds antimicrobial cooking losses reported in Table 1 (from 0 to 14.6%), resulting in a concentration effect. Secondly, two sulfonamides (sulfaguanidine and sulfamerazine) and three tetracyclines (oxytetracycline, chlortetracycline, and 4-epi-tetracycline) showed no significant variation in concentration during cooking with processing factors between 0.9 and 1.3. For oxytetracycline, chlortetracycline, and 4-epi-tetracycline, this can be explained by the fact that the antimicrobial cooking losses reported in Table 1 (from 25.6% to 37.0%) were of the same order of magnitude as the mean weight loss of ground beef patties during medium cooking (34.0%). The antimicrobial cooking losses of these three hydrophilic compounds (log Kow in the −1.3–0.44 range) may be explained, at least in part, by their expelling with water into cooking juice. For sulfaguanidine and sulfamerazine, the high variability of the results (RSD > 10%) may explain why there is no significant difference between their concentrations before and after cooking. Thirdly, a decrease in concentration (*p* < 0.05) could be observed in Table S1 for sulfamethoxazole and 4-epi-chlortetracycline with processing factors of 0.8 and antimicrobial cooking losses (44.6 ± 1.4% and 43.1 ± 1.4%, respectively) that exceed the weight loss of ground beef patties during medium cooking (34.0%) (Table 1). Although the matrix studied and the cooking mode were different, these last results are in line with those of Ismail-Fitry et al. [18] for a deep-fried chicken meat dish, in which average sulfamethoxazole losses were 40.7%, confirming that this sulfonamide is sensitive to the cooking process. In contrast, Furusawa et al. [17] reported slight differences between sulfonamides when roasting chicken thigh muscles for 12 min at 170 °C, with 38% loss of sulfaquinoxaline (none in our study), 39% of sulfamethoxazole (45% in our study), and 40% of sulfamonomethoxine (none in our study). For some tetracyclines, Abou Raya et al. [19] found greater antimicrobial losses than in our study after roasting chicken thigh meat for 40 min at 180 °C, with 61% loss of oxytetracycline (29% in our study), 58% loss of tetracycline (6% in our study), and 43% loss of chlortetracycline (37% in our study). Although no supporting data are available, these differences can be tentatively explained by greater quantities of juice expelled under the conditions used by Abou Raya et al. with a longer cooking time (40 min vs. 14 min in the present study), thus carrying off more hydrophilic tetracyclines.

All the results presented above were achieved with intentionally contaminated (spiked) samples in order to control the matrix composition and its antimicrobial load (spiking concentration of 200 µg.kg^−1^ of fresh meat, corresponding to twice the maximum residue limits (MRLs) [28]). To assess the scope of the conclusions drawn from spiked model samples, the same domestic medium pan cooking was applied to naturally contaminated (incurred) samples containing antimicrobials bioaccumulated at ultra trace concentrations during animal breeding. Incurred beef (*n* = 3) and pork (*n* = 5) muscles were sampled in the frame of the French project SOMEAT (www.so-meat.fr (accessed on 7 August 2022)). Tables S2 and S3 (Supplementary Material) give concentrations of the three sulfonamides (sulfadiazine, sulfamethazine, and sulfadimethoxine) and the three tetracyclines (tetracycline, oxytetracycline, and 4-epi-tetracycline) detected in these raw or medium-cooked samples. An increase in the concentration of sulfonamides and tetracyclines was observed in all studied samples because of the weight losses of meat samples that exceeded the antimicrobial losses reported in Tables S2 and S3. Antimicrobial cooking losses for incurred meat are of the same order of magnitude as those obtained with spiked meat for sulfadiazine (no loss vs. 3.5% loss for spiked meat), sulfamethazine (no loss for the two types of meat), sulfadimethoxine (0–2.4% loss vs. 0.3% loss for spiked meat), and tetracycline (0–17.3% loss vs. 5.9% loss for spiked meat). Tables S2 and S3 show that antimicrobial cooking losses observed with incurred samples are more variable than in spiked samples whose matrix composition and antimicrobial load are controlled. This confirms the importance of working on spiked samples in order to obtain mean robust and reproducible data that will allow clear conclusions to be drawn regarding the effect of cooking on antimicrobials. It is important to note that, for oxytetracycline and 4-epi-tetracycline, lower antimicrobial cooking losses for incurred samples were observed compared with spiked samples: 2.8–12.1% loss for oxytetracycline (28.9% for spiked meat) and 0–13.5% loss for 4-epi-tetracycline (25.6% for spiked meat). This suggests that the spiking step may not always reproduce identically the relationship that exists in incurred samples between antimicrobials and matrix components, resulting in greater antimicrobial losses during cooking. In incurred samples, tetracyclines may have crossed cell membranes and readily entered cells [29], while this phenomenon probably cannot be reproduced with spiking. This could explain why tetracyclines found in incurred samples are less expelled from the meat during cooking compared with spiked samples. However, the limited number of incurred samples available in this study does not allow to confirm this trend, and more extensive investigations will be necessary to elucidate the significance and mechanisms responsible for the observed differences between spiked and incurred samples.

### 2.2. Thermal Degradation of Sulfamethoxazole during Meat Cooking

To determine whether sulfonamide and tetracycline losses observed during meat cooking could be the consequence of thermal degradation, a proof of concept approach was carried out on the most cooking-sensitive of these compounds—sulfamethoxazole (Table 1). Its fate during cooking was investigated by use of radiolabeling associated with both radiodetection and HRMS analysis to obtain information on both the absolute quantification and the structural identification of degradation compounds. The impact of the cooking intensity (rare, medium, or well-done meat) on sulfamethoxazole thermal degradation was also investigated.

A protocol was first set up for radiolabeled sulfamethoxazole in order to study its thermal degradation (Figure S1, Supplementary Material). ^14^C-sulfamethoxazole recovery rates were determined after the spiking, extraction, purification, concentration, and analysis protocol. Mean recovery rates were 77.4 ± 14.6% for rare meat, 58.9 ± 7.3% for medium-cooked meat, and 55.4 ± 5.3% for well-done meat (*n* = 3 for each cooking level). Only mean recovery rates obtained with rare meat lay in the classically accepted range of 70–130%, according to the EPA Method 8000C. For medium-cooked and well-done meat, the low recovery rates obtained can be explained by the fact that protein denaturation occurs during cooking, such that the tissues shrink and then become harder and more compact when cooking intensity increases [30], thus limiting the efficiency of solvent extraction. For these cooking levels, the quantitative data obtained in this study must therefore be interpreted with caution.

**Figure 1:**
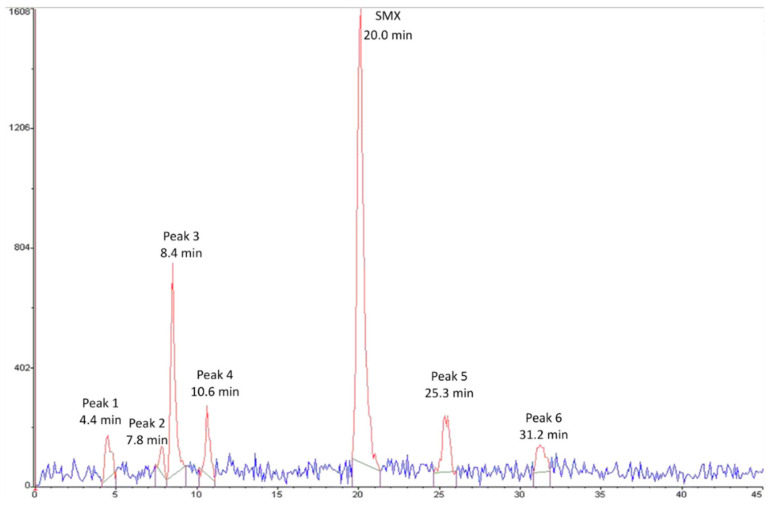
Typical radio-HPLC chromatogram of an extract of well-done cooked meat spiked with ^14^C-sulfamethoxazole (SMX). Six potential sulfamethoxazole breakdown products (Peak 1 to 6) can be detected.

Thanks to separation by HPLC and monitoring by online radioactivity detection, six potential degradation products of ^14^C-sulfamethoxazole were detected in cooked meat extracts (Figure 1). Table 2 displays the percentage of the radioactivity detected in meat extracts represented by each peak according to the cooking level. Whatever the cooking level, the radioactivity detected in meat extract remains mainly because of ^14^C-sulfamethoxazole (from 60% to 79% of the radioactivity detected). Peak 3 represents the major degradation compound (17% of the radioactivity detected in well-done meat). It can be found in trace amounts in raw meat, suggesting that this compound can appear during meat storage, but its formation significantly increased (*p* < 0.05) with cooking intensity. Peak 1 and Peak 4 appeared after medium and intense cooking but were not detected in rare meat, whereas Peak 2 appeared in trace amounts only after intense cooking (0.6% of the radioactivity detected in well-done meat). Finally, Peak 5 and Peak 6 are similarly found in raw and cooked meat, suggesting that these compounds appeared during meat storage but not during meat cooking. These results suggest that the 45% losses of sulfamethoxazole observed during medium cooking (Table 1) may be due, at least in part, to thermal degradation processes. These significant antimicrobial losses may also be explained by a release of sulfamethoxazole into the cooking juice. However, although the cooking juice was collected in this study, the quantitative results obtained after analysis were not sufficiently reproducible to be considered reliable and explainable in order to confirm this hypothesis.

### 2.3. Structural Identification of Sulfamethoxazole Thermal Degradation Products

**Figure 2:**
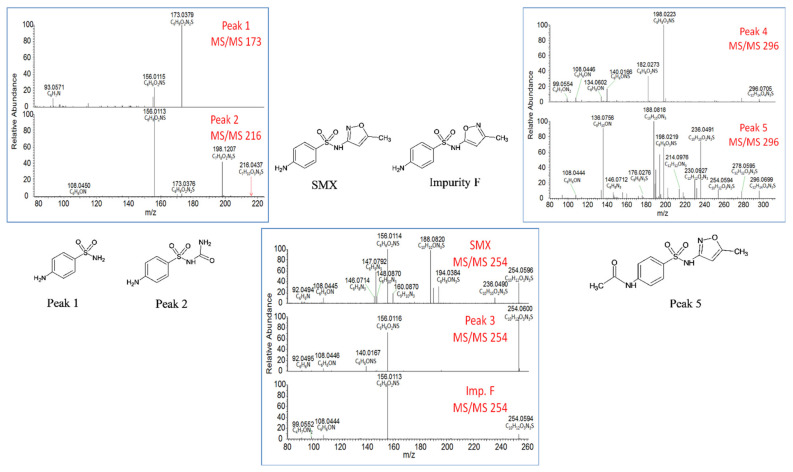
Fragmentation mass spectra of sulfamethoxazole (SMX) degradation compounds.

**Figure 3:**
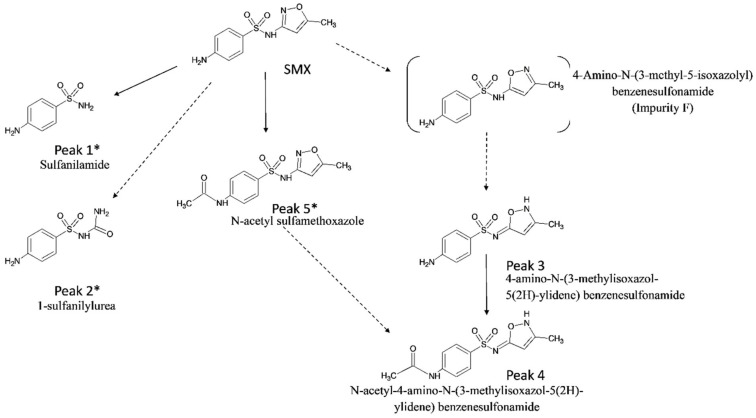
Proposed degradation scheme of sulfamethoxazole (SMX) during meat cooking. (* identity confirmed by comparison of chromatographic retention time and MS/MS spectra with an available standard compound).

Structural identification of the degradation compounds was carried out using MS and MS/MS experiments and high-resolution exact mass measurements on both molecular species and fragment ions. Protonated molecular ions were detected at *m/z* 173.0379 for Peak 1 (C_6_H_9_O_2_N_2_S, −2.3 ppm), *m/z* 216.4037 for Peak 2 (C_7_H_10_O_3_N_3_S, −1.8 ppm), *m/z* 254.0595 for Peak 3 (C_10_H_12_O_3_N_3_S, −0.8 ppm), *m/z* 254.0596 for SMX (C_10_H_12_O_3_N_3_S, −0.4 ppm), and *m/z* 296.0699 for both Peak 4 and Peak 5 (C_12_H_14_O_4_N_3_S, −1.0 ppm). Peak 6 gave no response under our analytical conditions and was not investigated further. The MS/MS spectra of sulfamethoxazole (SMX), Peaks 1, 2, and 3 all displayed a common diagnostic fragment ion at *m/z* 156.0115 corresponding to the sulfonyl aniline ion (Figure 2), indicating that for these three degradation compounds, this part of the molecule was not modified. Peaks 4 and 5 displayed a characteristic fragment ion at *m/z* 198.0223 (42 amu shift from the *m/z* 156 ion) corresponding to the N-acetyl sulfonyl aniline ion (Figure 2). For these two compounds, the amine function of the aniline moiety of SMX was therefore acetylated. On the basis of these results, SMX was thus shown to undergo thermal degradation into polar compounds, namely sulfanilamide (Peak 1) and 1-sulfanilylurea (Peak 2), whose identities were confirmed according to chromatographic retention time (Rt) and spectral data identical to those of standard compounds. These two compounds are formed by opening or breakage of the parent SMX oxazole moiety and have already been identified as SMX photodegradation products in water [31,32], but they are described for the first time in cooked meat. The major degradation compound (Peak 3) corresponded to an isomerization product from the initial 3-isoxazole form of SMX. By comparison of Rt and MS/MS spectra with that of an available standard compound, the conversion into the 3-methyl-5-isoxazolyl isomeric form of SMX (commercially so-called “impurity F”, Figure 3) could be ruled out. However, fragmentation mass spectra of Peak 3 (Figure 2) were not informative enough to provide the precise structure of this compound.

Therefore, further investigations using 1D and 2D (^1^H, ^1^H-^1^H COSY, ^1^H-^13^C HSQC, ^1^H-^13^C HMBC) NMR experiments were undertaken after peak collection for purification (Supplementary Material: NMR analyses). Analyses of the spectra obtained for Peak 3, as well as other SMX structurally related standard compounds, indicated that Peak 3 corresponded to 4-amino-N-(3-methylisoxazol-5(2H)-ylidene) benzenesulfonamide. Detailed results are reported in Supplementary Material. To the best of our knowledge, although SMX isomerization has already been reported (particularly in the frame of photodegradation in water [31,32,33]), this compound has never been described as a degradation product of SMX. Peak 5 was identified as the N-acetylated derivative of SMX (N-acetyl sulfamethoxazole, comparison with the available corresponding standard compound). N-acetylation is already known as a metabolic pathway of SMX [34]. By similarity, Peak 4 was identified as the N-acetylated derivative of Peak 3, although no reference standard compound was available for confirmation. On the basis of these results, a degradation scheme of sulfamethoxazole during meat cooking is proposed in Figure 3. To our knowledge, there are no recent data regarding the toxicity of these potential degradation products of sulfamethoxazole. To go further, it will be interesting to undertake toxicological studies in order to assess the potential toxicity of these compounds.

## 3. Materials and Methods

### 3.1. Chemicals and Standards

Methanol, acetonitrile, and ethanol were HPLC grade (Sharlau, Barcelona, Spain). Heptafluorobutyric acid 99.5%, pentafluoropropionic acid, hexane, formic acid, and diatomaceous earth used for the preparation of ASE cells were from Sigma-Aldrich (Saint-Quentin-Fallavier, France). Ultrapure water was obtained using a Milli-Q system (Millipore, Molsheim, France).

All nonradiolabeled sulfonamides and tetracyclines standards (purity > 95%) were from Sigma-Aldrich (Saint-Quentin Fallavier, France), except for 4-Epi-tetracycline hydrochloride and Epi-oxytetracycline chlorhydrate which were from VWR International (Fontenay-sous-Bois, France).

For HPLC-MS/MS analysis, sulfadimethoxine-D_6_, sulfadiazine-^13^C_6_, sulfathiazole-^13^C_6_, sulfadimidine-^13^C_6,_ and sulfadoxine-D_3_ and demeclocycline hydrochloride used as internal standards were purchased from Witega (Berlin, Germany), and from Sigma-Aldrich (Saint-Quentin Fallavier, France), respectively. Trichloroacetic acid, ethylenediaminetetraacetic acid trisodium salt trihydrate (EDTA), and citric acid monohydrate were from Fisher Scientific (Illkirch, France). Glacial acetic acid and oxalic acid dehydrate were from VWR International (Fontenay-sous-Bois, France). Anhydrous disodium hydrogen phosphate (Na_2_HPO_4_) was from Roth Sochiel (Lauterbourg, France).

For radio-HPLC analysis, ^14^C-Sulfamethoxazole (specific activity of 77 mCi.mmol^-1^) was obtained from American Radiolabeled Chemicals Inc. (St Louis, MO, USA). Radiopurity was checked by radio-HPLC, and the chemical purity obtained for this standard was greater than 99%. Stock solutions were prepared in ethanol and kept at −20 °C. Flo-Scint II and Ultima Gold liquid scintillation cocktails were purchased from Perkin Elmer Life and Analytical Sciences (Courtaboeuf, France).

For NMR analysis, sulfamethoxazole, sulfanilamide, sulfamethoxazole impurity F, and 1-sulfonylurea were purchased from Sigma Aldrich. N-acetyl sulfamethoxazole was purchased from Cayman Chemical (Ann Arbor, MI, USA).

### 3.2. Meat Samples

Two types of meat samples were used: intentionally contaminated (*n* = 3 for each cooking condition) and naturally incurred samples (*n* = 8). In order to work on a homogeneous matrix uniformly contaminated at a known and high enough concentration to be detected, intentionally contaminated meat was prepared with ground beef samples from the same blend of muscles (11% fat) purchased from a French supplier. The total of 125 g weight aliquots was stored at −80 °C before use. Matrix blank aliquots of these samples were made before spiking.

Naturally incurred meats were sampled in the frame of the French project SOMEAT (Contract No. ANR-12-ALID-0004. Safety of Organic Meat. Available at www.so-meat.fr (accessed on 7 August 2022)). These samples were ground before use, similar to intentionally contaminated.

### 3.3. Meat Spiking

The spiking method of combining micropollutants addition to ground meat with matrix homogenization was carried out according to Planche et al. [24]. For nonradiolabeled sulfonamides and tetracyclines, a spiking concentration of 200 µg.kg^−1^ of fresh meat, corresponding to twice the maximum residue limits (MRLs) [28], was chosen, whereas, for radiolabeled sulfamethoxazole, meat was spiked at a concentration of 0.019 µCi.g^-1^ of fresh meat. Small ground beef patties weighing 26 g (1.5 × 4.5 × 4.5 cm) were then shaped to mimic commercial ground beef patties. Samples were then stored at −80 °C before analysis under dark conditions.

### 3.4. Cooking Method

To study the impact of a standardized and reproducible domestic cooking method on sulfonamides and tetracyclines, ground beef patties were cooked in a stainless steel frying pan (17 cm diameter) on a controlled-temperature induction hob (Bosch Electroménager, Saint-Ouen, France), according to Planche et al. [24]. A sheet of 11 µm thick aluminum foil was laid on the bottom of the frying pan to recover juice released during meat cooking. Three different cooking conditions were used to simulate rare (core 50 °C), medium (core 70 °C, required temperature for the inactivation of pathogens [35]), or well-done (core 85 °C) meat (*n* = 3 for each cooking condition). These cooking conditions corresponded to 7 min heating (patty turned over once) at 160 °C at the bottom of the pan, 14 min heating (turned over three times) at 200 °C at the bottom of the pan, and 14 min heating (turned over three times) at 250 °C at the bottom of the pan, respectively (Figure S3, Supplementary Material). Temperatures at the core of the meat and at the bottom of the pan were continuously monitored during cooking by thermosensors (RS Components, Beauvais, France).

### 3.5. Determination of Antimicrobial Residues in Raw and Cooked Meat

Extractions and analyses were performed in line with the analytical methods developed at the French National and European Union Reference Laboratory (EURL) in charge of Veterinary Medicinal Product Residues and Dye residues in Food from Animal Origin (Fougères Laboratory, ANSES-Fougères, France). The two implemented methods for national monitoring and control of sulfonamides (F/CHIM/SM/PTC/016) and tetracyclines (F/CHIM/SM/PTC/007) in raw meat of muscle were previously validated according to Commission Decision 2021/808/EC and accredited by COFRAC under standard ISO17025 [36,37].

#### 3.5.1. Sample Extraction

For the analysis of the 15 sulfonamides, the extraction procedure consisted of weighing 2 g of ground beef sample (raw or cooked) followed by the addition into the samples of 200 µL of internal standard (isotopically labeled sulfonamide standards) solution at 1 µg.mL^−1^ and 200 µL of ultrapure water. Then, samples were extracted with 8 mL of acetonitrile, rotary-homogenized at 100 rpm for 10 min before centrifugation at 14,000× *g* at 4 °C for 5 min. Then, 6 mL of resulting supernatant were transferred into a clean sample tube and were evaporated to dryness at 50 °C. The extracts were dissolved in 1 mL of ultrapure water and filtered through a 0.45 µm syringe filter before LC-MS/MS analysis.

The preparation of raw and cooked samples for the analysis of the six tetracyclines consisted of weighing 2 g of meat, followed by the addition of 200 µL of a 1 µg.mL^−1^ demeclocycline (internal standard) solution and 800 µL of ultrapure water. Then, 10 mL of Mac Ilvaine/EDTA solution was added to samples and rotary-homogenized at 100 rpm for 10 min before centrifugation at 14,000× *g* at 4 °C for 10 min. Deproteinization of samples was then carried out by adding 1 mL of trichloroacetic acid at 1g.mL^−1^; samples were vortexed for 10 s and stored at −20 °C for 15 min. Samples were then centrifuged at 14,000× *g* at 4 °C for 5 min, and the supernatants were sampled and loaded onto preconditioned Solid Phase Extraction cartridges (Bond-Elut). After the cleaning of the cartridge with 1 mL of ultrapure water and 5 min drying of the cartridge, elution was performed with 2 × 0.6 mL of an oxalic acid solution in methanol at 0.01 mol.L^−1^ and 1.8 mL of ultrapure water. Extracts were then filtered through a 0.45 µm syringe filter before LC-MS/MS analysis.

#### 3.5.2. Analysis by HPLC-MS/MS

For the HPLC-MS/MS analysis of sulfonamides, 10 µL of the sample extract was injected into an LC-20ADXR system (Shimadzu, Marne la Vallée, France) for the chromatographic separation step using an RP C18 Symmetry column (100 mm × 2.1 mm i.d., 3.5 µm particle size, Waters, Saint-Quentin en Yvelines, France) with a security guard column C18 (4.0 mm × 2.0 mm i.d., Phenomenex, Le Pecq, France). Elution gradient was performed at a constant flow rate of 250 µL.min^−1^ and with a binary mobile phase: ultrapure water (A) and acetonitrile (B), each solvent containing 1 mmol.L^−1^ heptafluorobutyric acid. The elution gradient started with 10% of B and linearly rose to 30% of B during the 4th min. Then B was held at 30% for 1 min before a linear raising to 70% over 2 min, then held at 70% over the next 3 min, returned to the initial condition (a linear gradient back to 10% over 1 min), and held at 10% over additional 3 min for a re-equilibration time.

Detection and identification of the sulfonamides of interest were performed on an API 5500 triple quadrupole mass detector (AB-Sciex, Les Ulis, France). The electrospray ionization source operated in the positive mode, and the following parameters were tuned: source temperature was set at 650 °C, the curtain gas set at 20 psi, the ion spray voltage set at 4000 V, and the ion source gas 1 and 2 set at 25 psi and 30 psi, respectively. The scan mode for MS/MS detection was set on scheduled Multiple Reaction Monitoring (MRM).

For the HPLC-MS/MS analysis of tetracyclines, 25 µL of the sample extract was injected into an LC-20ADXR system (Shimadzu, Marne-la-Vallée, France) for the chromatographic separation step using an RP C18 Symmetry column (100 mm × 2.1 mm i.d., 3.5 µm particle size, Waters, Saint-Quentin en Yvelines, France) with a security guard column C18 (4.0 mm × 2.0 mm i.d., Phenomenex, Le Pecq, France). Separation was performed at a flow rate of 300 µL.min^−1^ during a runtime analysis of 20 min with a binary mobile phase as follows: ultrapure water + 0.1% pentafluoropropionic acid (A) and acetonitrile (B). The elution gradient started with 15% of B and linearly rose to 50% during the seventh minute. Then B was held at 50% for 2 min before a linear gradient to return to initial conditions and held at 15% over 10 min for a re-equilibration time.

Detection and identification of the tetracyclines of interest were performed on an API 5500 triple quadrupole mass detector (AB-Sciex, Les Ulis, France). The electrospray ionization source operated in the positive mode, and the following parameters were tuned: source temperature was set at 500 °C, the curtain gas was set at 20 psi, the ion spray voltage was set at 4000 V, and the ion source gas 1 and 2 at 40 psi and 50 psi, respectively. The scan mode for MS/MS detection was set on scheduled Multiple Reaction Monitoring (MRM). The presence of internal standards was checked in all samples. Identification was performed according to the Commission Decision 2021/808/EC.

Quantification of sulfonamides and tetracyclines in raw samples and cooked samples was processed thanks to a calibration curve which was prepared from blank raw muscle samples spiked at different levels of concentration. Quality control samples of spiked blank samples of cooked muscle were added to the sequence of analysis in order to check the validity of the analysis of cooked samples.

### 3.6. Identification and Quantification of Sulfamethoxazole Degradation Products Using Radiolabeling

#### 3.6.1. Extraction of ^14^C-Sulfamethoxazole and Its Degradation Products

Before extraction, the cooked ground beef patties were reduced to powder using a liquid nitrogen grinder, then extracted by accelerated solvent extraction (ASE) using a Dionex ASE 350 extractor (Sunnyvale, CA, USA) with 34 mL stainless-steel extraction cells. The cells were filled with 5 g of ground beef dispersed in diatomaceous earth. Paper filters were placed at the bottom and at the top of the extraction cell. According to Hoff et al. [38], prior to extraction, the cells were submitted to a cleanup method in order to remove the lipids from the samples by using hexane as solvent. ASE conditions were as follows: temperature 60 °C, two cycles of 5 min each, 5 min static time, and pressure 1500 psi. The total flush volume of 80% and 300 s purge time with nitrogen flow were applied. At the next step, the same ASE cells containing the samples were submitted to extraction using acetonitrile as extraction solvent set at a temperature of 90 °C and a pressure of 1500 psi. Three cycles of 5 min each were carried out. A total flush volume of 80% and 60 s purge with nitrogen flow were applied. The acetonitrile extract was then evaporated, first in a “Rotavapor” apparatus and second under nitrogen flow until dryness. Extracts were redissolved in 500 µL of mobile phase mixture (95% water/5% acetonitrile +0.2% formic acid) and transferred to a HPLC vial.

Radioactivity losses were checked at each step of the protocol by direct counting on a liquid scintillation analyzer (Tri-Carb 2910 TR/PerkinElmer, France), using Ultima Gold (PerkinElmer) as a scintillation cocktail.

#### 3.6.2. Quantification of ^14^C-Sulfamethoxazole Degradation Products

^14^C-Sulfamethoxazole and its degradation products were analyzed on an Agilent 1200 (Agilent Technologies, Les Ulis, France) liquid chromatograph connected to a Flo-one/β A500 instrument (Radiomatic, La-Queue-Lez-Yvelines, France), with Flow-scint II as scintillation cocktail (Packard Instruments). The HPLC system consisted of a C18 Nucleodur column (250 × 4 mm, 5 μm; Macherey Nagel) maintained at a temperature of 35 °C. Conditions were as follows: mobile phases consisted of (A) a mixture of water/acetonitrile 95/5 *v/v* with 0.2% formic acid and (B) pure acetonitrile. The flow rate was 1 mL.min^−1^. The following gradient was used: 0–2 min 100% A; 2–4 min linear gradient from 0% to 12% B; 4–27 min linear gradient from 12% to 15% B; then 27–30 min linear gradient from 15% to 100% B, held for 10 min. Degradation compounds were quantified by integrating the area under the peaks monitored by radioactivity detection.

#### 3.6.3. Structural Characterization of ^14^C-Sulfamethoxazole Degradation Products

For structural characterization of the degradation compounds, analyses were performed by HPLC coupled to an LTQ Orbitrap XL hybrid high-resolution mass spectrometer (Thermo Scientific, Les Ulis, France) fitted with an electrospray ionization source operating in the positive mode, using the HPLC conditions described above for radio-HPLC profiling, and a 1/4 postcolumn splitting. Typical operating parameters used for ion production were as follows: spray voltage (4.5 kV), sheath gas (N_2_) flow rate (35 arbitrary units (au)), auxiliary gas (N2) flow rate (5 au), heated transfer capillary temperature (350 °C), heated transfer capillary voltage (20 V), and tube lens voltage (75 V). High-resolution mass spectra were acquired from *m/z* 80 to 600 at a resolution power of 60,000. Identifications were performed by tandem MS experiments using the ion-trap mass analyzer of the LTQ Orbitrap spectrometer. For MS/MS experiments, resolution power was reduced to 7500, and the excitation parameters (isolation width, normalized collision energy, and excitation time) were adjusted to obtain the maximum structural information for the compound of interest. All analyses were achieved under automatic gain control conditions using helium as damping as well as collision gas for MS/MS experiments. NMR spectra were recorded at 300K on a Bruker Avance III HD spectrometer (Bruker) fitted with a 5 mm CQPCI-cryoprobe operating at 600.13 MHz. NMR tubes of 3 mm were used. Chemical shifts are reported in parts per million (ppm) relative to DMSO-d6 (2.54 ppm/40.55 ppm).

### 3.7. Data Processing

Antimicrobial losses induced by cooking were determined according to Rawn et al. [26]:

(1) $$\mathrm{Antimicrobial}\;\mathrm{loss}=1-\frac{\left(\left[Antimicrobial\;cooked\;meat\right]\times mass\;cooked\;meat\right)}{\left(\left[Antimicrobial\;raw\;meat\right]\times mass\;raw\;meat\right)}$$

where [*Antimicrobial raw meat*] and [*Antimicrobial cooked meat*] are the antimicrobial measured concentrations in raw or cooked meat (µg.kg^−1^ of meat) and mass raw meat and mass cooked meat are the mass of meat (g) before and after cooking.

Processing factors (PF) were determined according to Saber et al. [27]:

(2) $$\mathrm{PF}=\frac{\left[Antimicrobial\;cooked\;meat\right]}{\left[Antimicrobial\;raw\;meat\right]}$$

A processing factor value of <1 (=reduction factor) indicates a reduction of the antimicrobial residues in the cooked meat, whereas a value of >1 (=concentration factor) indicates a concentration effect of cooking.

Data were processed using Statistica software version 10 (StatSoft, Maisons-Alfort, France). Student’s *t*-test or one-way analyses of variance (ANOVA) followed by posthoc Newman–Keuls test was performed to compare means data. The differences were considered significant when *p* < 0.05.

## 4. Conclusions

The fate of 15 sulfonamides and 6 tetracyclines during meat cooking was assessed. Results show that for 12 sulfonamides and 2 tetracyclines, cooking induces a concentration effect. In contrast, domestic cooking can induce a significant decrease in levels of 4-epi-chlortetracycline (43% loss) and sulfamethoxazole (45% loss). A proof of concept approach carried out on the most cooking-sensitive of these compounds (sulfamethoxazole) demonstrated that antimicrobial losses could be the consequence of thermal degradation of antimicrobials during cooking. Among the identified degradation products of sulfamethoxazole, sulfanilamide and N-acetyl sulfamethoxazole appeared after medium and intense cooking, sulfonylurea appeared only after intense cooking, and 4-amino-N-(3-methylisoxazol-5(2H)-ylidene) benzenesulfonamide can appear during meat storage, but its formation significantly increased with cooking intensity. This study highlights the importance of taking into account the cooking step in chemical risk assessment procedures considering its impact on the level of chemical contaminants in meat and on the formation of potentially toxic breakdown compounds. In order to go further, it will be interesting to extend this research to other food chemical contaminants and to undertake toxicological studies in order to assess the potential toxicity of their cooking degradation products.

## Figures and Tables

**Table 1:** Losses of sulfonamides and tetracyclines during medium cooking and corresponding processing factors. Antimicrobial losses were determined according to Rawn et al. [26], considering the mass of meat before and after cooking. Concentrations of sulfonamides and tetracyclines in raw and medium-cooked meat are reported in Table S1 (Supplementary Material).

Compound	Antimicrobial Cooking Loss (%)	Mean Processing Factor (PF)
Sulfaguanidine ^2^	21.7 ± 1.9	1.2
Sulfacetamide ^1^	3.3 ± 2.4	1.4 *
Sulfadiazine ^1^	3.5 ± 2.4	1.4 *
Sulfamethoxazole ^3^	44.6 ± 1.4	0.8 *
Sulfathiazole ^1^	9.4 ± 2.2	1.3 *
Sulfamerazine ^2^	14.7 ± 2.1	1.3 *
Sulfamethizole ^1^	None	1.6 *
Sulfamethazine ^1^	None	1.5 *
Sulfamethoxypyridazine ^1^	1.2 ± 2.4	1.5 *
Sulfamonomethoxine ^1^	None	1.5 *
Sulfaquinoxaline ^1^	None	1.6 *
Sulfadoxine ^1^	6.2 ± 2.3	1.4 *
Sulfadimethoxine ^1^	0.3 ± 2.4	1.5 *
Sulfaclozine ^1^	None	1.5 *
Sulfachloropyridazine ^1^	4.5 ± 2.3	1.4 *
Tetracycline ^1^	5.9 ± 2.3	1.4 *
Doxycycline ^1^	14.6 ± 2.1	1.3 *
Oxytetracycline ^2^	28.9 ± 1.7	1.1
Chlortetracycline ^2^	37.0 ± 1.5	0.9
4-epi-Tetracycline ^2^	25.6 ± 1.8	1.1
4-epi-Chlortetracycline ^3^	43.1 ± 1.4	0.8 *

* Significant differences in antimicrobial concentrations between raw and medium cooked meat. ^1^ Significant increase in concentration during cooking (*p* < 0.05) with processing factors ≥1.3. During medium cooking, the mean weight loss of meat (34.0%) exceeds losses of these compounds. ^2^ No significant variation in concentration during cooking with processing factors between 0.9 and 1.3. For oxytetracycline, chlortetracycline, and 4-epi-tetracycline, antimicrobial cooking losses are of the same order of magnitude as the mean weight loss of meat during medium cooking (34.0%). For sulfaguanidine and sulfamerazine, the high variability of the concentrations presented in Table S1 does not allow us to obtain reliable data (*p* > 0.05). ^3^ Significant decrease in concentration during cooking (*p* < 0.05) with processing factors ≤ 0.8. During medium cooking, losses of these compounds exceed the mean weight loss of meat (34.0%).

**Table 2:** Percentage of the radioactivity detected by Radio-HPLC represented by each peak according to cooking level (*n* = 3 for each cooking level). Six peaks corresponding to potential degradation products of ^14^C-sulfamethoxazole (SMX) were detected in raw and cooked meat extracts.

	Raw Meat	Rare Meat	Medium-Cooked Meat	Well-Done Meat
SMX	76.1 ± 5.8 ^a,b^	78.6 ± 1.7 ^b^	72.0 ± 2.2 ^a^	59.6 ± 3.9 ^a^
Peak 1	n.d. ^a^	n.d. ^a^	3.0 ± 2.2 ^b^	4.8 ± 1.9 ^c^
Peak 2	n.d. ^a^	n.d. ^a^	n.d. ^a^	0.6 ± 1.1 ^a^
Peak 3	1.9 ± 3.3 ^a^	6.2 ± 2.1 ^a,b^	12.7 ± 4.0 ^a,b^	17.0 ± 10.3 ^b^
Peak 4	n.d. ^a^	n.d. ^a^	1.2 ± 1.4 ^a,b^	2.7 ± 2.3 ^b^
Peak 5	8.1 ± 4.4 ^a,b^	7.9 ± 3.3 ^b^	6.1 ± 3.4 ^a^	8.0 ± 3.1 ^a,b^
Peak 6	13.9 ± 4.6 ^b^	7.3 ± 1.4 ^a,b^	5.1 ± 3.6 ^a^	7.6 ± 6.3 ^a,b^

^a,b,c^—different superscript letters within the same row indicate significant differences among values (*p* < 0.05). n.d.—non detected.

## Data Availability

Data presented in this paper are available from the authors.

## Supplementary Materials

The following supporting information can be downloaded at https://www.mdpi.com/article/10.3390/molecules27196233/s1, Figure S1: experimental flowchart used to analyze ^14^C-sulfamethoxazole and its cooking degradation products in spiked meat, Table S1: concentrations of sulfonamides and tetracyclines in raw and medium cooked meat, Table S2: concentrations of tetracycline, oxytetracycline and 4-epi-tetracycline in raw and medium-cooked incurred beef and pork samples, and corresponding cooking losses, Table S3: concentrations of sulfadiazine, sulfamethazine, and sulfadimethoxine in raw and medium-cooked incurred pork samples, and corresponding cooking losses; Table S4, Figure S1 and Figure S2: NMR analyses, Figure S3: cooking conditions used to simulate rare, medium, or well-done meat. Ref [26,33,39,40,41] are cited in Supplementary Materials.

## Sample Availability

Samples of the compounds described herein are available from the authors.
