# Supplementary material for: Fate of Sulfonamides and Tetracyclines in Meat during Pan Cooking: Focus on the Thermodegradation of Sulfamethoxazole
Source: Molecules. 2022 Sep 22;27(19):6233. doi: 10.3390/molecules27196233 (PMC9571958; doi:10.3390/molecules27196233)
Supplement: Supplementary file 1 [file molecules-27-06233-s001.zip › molecules-1880303-supplementary.pdf]

Supplementary Material

Table S1. Concentrations of sulfonamides and tetracyclines in raw (*n* = 3) and medium (*n* = 3) cooked meat. The two implemented methods for national monitoring and control of sulfonamides (F/CHIM/SM/PTC/016) and tetracyclines (F/CHIM/SM/PTC/007) in raw meat of muscle were previously validated according to Commission Decision 2021/808/EC and accredited by COFRAC under standard ISO17025. Relative standard deviations (%) of concentrations are presented in brackets. a–b: different superscript letters within the same row indicate significant differences among values ($p<0.05$).

Compound	Concentration in raw meat (μg.kg^{-1} of meat)	Concentration in medium-cooked meat (μg.kg^{-1} of meat)
Sulfaguanidine[2]	180.7^{a} (5.7)	209.1^{a} (11.4)
Sulfacetamide[1]	147.7^{a} (6.9)	211.0^{b} (6.7)
Sulfadiazine[1]	163.9^{a} (6.8)	233.8^{b} (4.8)
Sulfamethoxazole[3]	155.1^{a} (5.0)	127.1^{b} (9.2)
Sulfathiazole[1]	154.0^{a} (20.0)	206.3^{b} (3.0)
Sulfamerazine[2]	191.9^{a} (20.0)	241.9^{a} (4.0)
Sulfamethizole[1]	150.2^{a} (6.6)	237.3^{b} (2.9)
Sulfamethazine[1]	159.4^{a} (4.8)	241.1^{b} (4.2)
Sulfamethoxypyridazine[1]	163.8^{a} (3.7)	239.1^{b} (2.2)
Sulfamonomethoxine[1]	158.7^{a} (4.9)	243.8^{b} (8.8)
Sulfaquinoxaline[1]	146.7^{a} (8.4)	228.9^{b} (7.4)
Sulfadoxine[1]	173.6^{a} (0.7)	240.7^{b} (4.3)
Sulfadimethoxine[1]	171.5^{a} (4.3)	252.6^{b} (6.0)
Sulfaclozine[1]	155.0^{a} (6.7)	239.3^{b} (7.0)
Sulfachloropyridazine[1]	155.4^{a} (5.9)	219.3^{b} (8.5)
Tetracycline[1]	222.8^{a} (3.7)	309.9^{b} (7.4)
Doxycycline[1]	211.4^{a} (4.1)	266.9^{b} (9.5)
Oxytetracycline[2]	227.8^{a} (3.9)	239.3^{a} (5.3)

Chlortetracycline[2]	220.5[a] (2.7)	205.2[a] (8.6)
4-epi-Tetracycline[2]	199.3[a] (3.4)	219.1[a] (6.2)
4-epi-Chlortetracycline[3]	188.7[a] (4.8)	158.6[b] (6.2)

[1]Significant increase in concentration during cooking ($p<0.05$) with processing factors ≥1.3

[2]No significant variation in concentration during cooking with processing factors between 0.9 and 1.3

[3]Significant decrease in concentration during cooking ($p<0.05$) with processing factors ≤0.8

Table S2. Concentrations (µg.kg^{-1} of meat) of tetracycline, oxytetracycline and 4-epi-tetracycline in raw and medium-cooked incurred beef and pork samples, and corresponding cooking losses determined according to Rawn *et al*. [26].

	Tetracycline			Oxytetracycline			4-epi-Tetracycline		
	Concentration in raw meat	Concentration in cooked meat	Cooking losses (%)	Concentration in raw meat	Concentration in cooked meat	Cooking losses (%)	Concentration in raw meat	Concentration in cooked meat	Cooking losses (%)
Sample 1 (Beef)	NA	NA	NA	223.5 ± 10.6	310.6 ± 5.9	2.8 ± 1.0	NA	NA	NA
Sample 2 (Beef)	36.8 ± 9.6	64.7 ± 4.2	None	NA	NA	NA	18.1 ± 5.8	34.9 ± 1.9	None
Sample 3 (Beef)	NA	NA	NA	327.0 ± 12.7	387.5 ± 10.6	12.1 ± 2.1	NA	NA	NA
Sample 4 (Pork)	129.5 ± 4.9	138.8 ± 0.4	17.3 ± 2.8	NA	NA	NA	69.3 ± 0.3	77.7 ± 1.1	13.5 ± 4.4
Sample 5 (Pork)	NA	NA	NA	418.8 ± 34.8	583.8 ± 18.7	5.8 ± 0.5	NA	NA	NA

NA: non-available (non-detected in meat extract)

Table S3. Concentrations (µg.kg^{-1} of meat) of sulfadiazine, sulfamethazine and sulfadimethoxine in raw and medium-cooked incurred pork samples, and corresponding cooking losses determined according to Rawn *et al*. [26].

	Sulfadiazine			Sulfamethazine			Sulfadimethoxine		
	Concentration in raw meat	Concentration in cooked meat	Cooking losses (%)	Concentration in raw meat	Concentration in cooked meat	Cooking losses (%)	Concentration in raw meat	Concentration in cooked meat	Cooking losses (%)
Sample 6 (Pork)	NA	NA	NA	NA	NA	NA	7.6 ± 0.3	11.0 ± 0.1	None
Sample 7 (Pork)	NA	NA	NA	51.1 ± 1.6	69.6 ± 2.0	None	62.8 ± 1.4	77.9 ± 0.3	2.4 ± 1.6
Sample 8 (Pork)	105.0 ± 1.3	133.5 ± 2.1	None	NA	NA	NA	NA	NA	NA

NA: non-available (non-detected in meat extract)

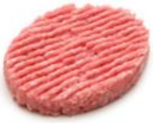

Ground beef

Spiking with ^{14}C-sulfamethoxazole
(40 000 dpm/g fresh meat)

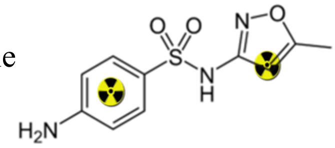

26 g of spiked meat

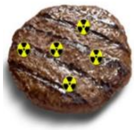

Pan cooking
(rare / medium / well-done cooked meat)

Liquid nitrogen grinding

5 g of cooked meat powder

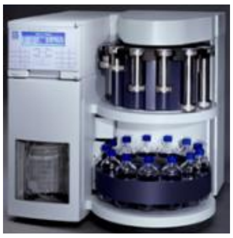

Accelerated solvent extraction:

- *First extraction with hexane (clean-up)*
- *Second extraction with acetonitrile (extraction of ^{14}C- sulfamethoxazole)*

Evaporation to dryness of the acetonitrile extract
(rotavapor and nitrogen flow)

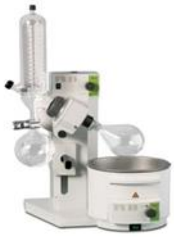

Addition of 500 µL of mobile phase
(95% water / 5% acetonitrile + 0.2% formic acid)

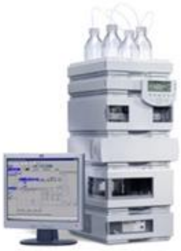

Radio-HPLC analysis
(Nucleodur C18 Gravity (250x4) mm, 5 µm; 35°C; 1 mL min^{-1})

Figure S1. Experimental flowchart used to analyze ^{14}C-sulfamethoxazole and its cooking degradation products in spiked meat.

NMR analyses

Experimental

NMR spectra were recorded at 300K on a Bruker Avance III HD spectrometer (Bruker) fitted with a 5mm CQPCI-cryoprobe operating at 600.13 MHz. 3 mm NMR tubes were used. Chemical shifts are reported in parts per million (ppm) relative to DMSO-d6 (2.54 ppm / 40.55 ppm).

Results

In order to elucidate the structure of peak 3, the following compounds were analyzed by NMR spectroscopy, using 1D and 2D experiments (^{1}H, ^{1}H-^{1}H COSY, ^{1}H-^{13}C HSQC, ^{1}H-1^{3}C HMBC):
Peak 3 after purification by HPLC and peak collection
Standard 4-amino-N-(5-methyl-1,2-oxazol-3-yl) benzenesulfonamide (SMX)
Standard 4-Amino-N-(3-methyl-5-isoxazolyl) benzenesulfonamide (described as SMX related Impurity F)
In addition, in the absence of any other related standard compound, data available from the literature were used for comparison with 4-Amino-N-(5-methyl-2-oxazolyl) benzenesulfonamide (described as degradation product 1 by Zhou *et al.* [1]).

Results are reported in Table 1. ^{1}H and ^{1}H-^{1}H COSY spectra of peak 3 show chemical shifts that are consistent with a structure containing a para-disubstituted phenyl ring and a heterocycle. Contrary to the three other compounds, no signal is detected for the NH bonded to the oxazole / isoxazole like ring in the ^{1}H spectrum of peak 3, suggesting that peak 3 could be an imido isomerized form of the oxazole / isoxazole ring. Indeed, sulfanilamide derivatives are known to exist as amido and imido tautomers [2].

Table S4: ^{1}H and ^{13}C NMR data of peak 3, impurity F, product 1^{a} and SMX (DMSO-d6)

	^{1}H				^{13}C			
	δ (ppm) (multiplicity, coupling constant)				δ (ppm) (multiplicity, coupling constant)			
	Peak 3	*Impurity F*	*product 1^{a}*	*SMX^{a}*	*Peak 3*	*Impurity F*	*product 1^{a}*	*SMX^{a}*
NH		11,7	11,3	10,9				
		(s)	(s)	(s)				
NH_2	5,33	5,64	5,79	6,06				
	(s)	(s)	(s)	(s)				
CH (heterocycle)	6,09	6,12	6,76	6,12	121,5	88,3	112,3	95,2
	(s)	(s)	(d, 8,5 Hz)	(d, 0,8 Hz)				
CH_3	2,03	2,11	2,08	2,28	11,4	12,3	11	12,2
	(s)	(s)	(s)	(s)				
C(heterocycle)-N					161,8		143,4	169,7
C(heterocycle)-Me					138,5	161,7	158,8	157,8
C1					134,4	124,5	130,9	124
CH 2,6	7,45	7,5	7,45	7,46	129,9	129,7	129,2	128,7
	(d, 8,5 Hz)	(d, 8,8 Hz)	(d, 8,7 Hz)	(d, 8,8 Hz)				
CH 3,5	6,47	6,62	6,53	6,57	114,8	113,6	114,2	112,5
	(d, 8,5 Hz)	(d, 8,8 Hz)	(d, 8,7 Hz)	(d, 8,8 Hz)				
C4					150,1	154,6	153,7	153,1
	a : Zhou W, Moore, DE : Int J Pharm, 1994, 110, 55-63							

^1H-^{13}C correlation spectra enable the assignment of ^{13}C chemical shifts. Since mass spectrometric data indicated that peak 3 must display an isomeric structure of the three other compounds, modifications of the heterocycle (i.e. isoxazole or oxazole ring, position of the methyl group on the heterocycle) were considered for peak 3.

The commercially available standard SMX and its related "Impurity F" (isoxazole ring differing from SMX by the position of the oxygen and nitrogen atoms on the heterocycle) were then analyzed. ^1H chemical shifts observed for SMX and "Impurity F" were comparable to those of peak 3, except the methyl and the 3,5-aromatic protons which are slightly shielded in peak 3. Yet, 2D experiments show important differences in carbon chemical shifts. In the heterocycle, both the carbon bearing the methyl group and the CH obviously differ between peak 3 and Impurity F. The chemical shift of C1 in the phenyl ring also reveals 10 ppm shift between the two compounds. All these data were in favor of an isomeric imido-stabilized structure for peak 3.

Since no standard compound was available for this structure, we turned to spectra simulations of SMX derivatives using nmrdb.org prediction tools [3,4]. Results of simulations are reported below in figure 1, where blue values correspond to simulated chemical shifts and black values to experimental ones when available. First, simulated and experimental spectra obtained for SMX, "Impurity F" and "product 1" were compared and the two data sets were found in very good agreement, showing that the prediction tools were reliable for these structures. The experimental data from peak 3 spectra were then compared to simulations of various imido forms of the heterocycle. As indicated in figure 1, only the simulated spectra obtained for the imido tautomeric form of impurity F matched very well the experimental spectra of peak 3.

From all these information, it was therefore concluded that peak 3 corresponded to the imido form of impurity F, i.e. 4-amino-N-(3-methylisoxazol-5(2H)-ylidene) benzenesulfonamide.

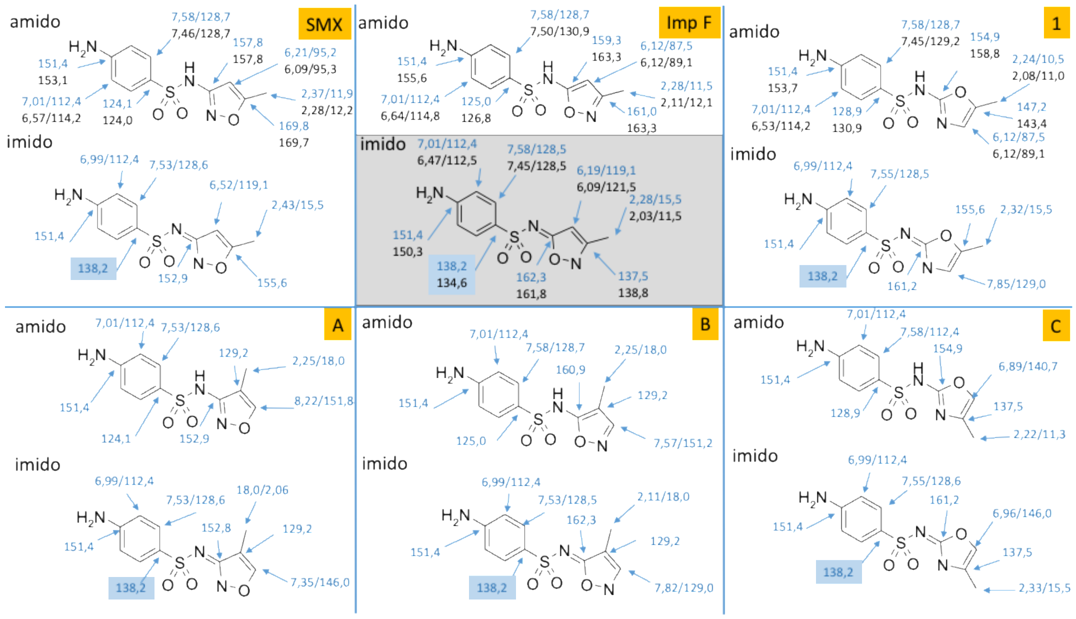

Figure S2: 1H and ^{13}C chemical shifts of various sulfamethoxazole related derivatives. In blue: nmr-db.org simulations; in black: experimental data.

1. Zhou W, Moore DE. Photochemical decomposition of sulfamethoxazole. *Int J Pharm* 1994, *110*, 55-63.
2. Bult A, Klasen HB. A spectrometric study of the tautomeric forms of some sulfanilamide derivatives in different media. *Pharm Weekbl* 1978, *113*, 665-672.
3. Andrés M. Castillo, Luc Patiny and Julien Wist. Fast and Accurate Algorithm for the Simulation of NMR spectra of Large Spin Systems. *J Magn Resonance* 2011, *209*, 123-130.
4. Aires-de-Sousa, M. Hemmer, J. Gasteiger, Prediction of 1H NMR Chemical Shifts Using Neural Networks, *Anal Chem* 2002, *74(1)*, 80-90.

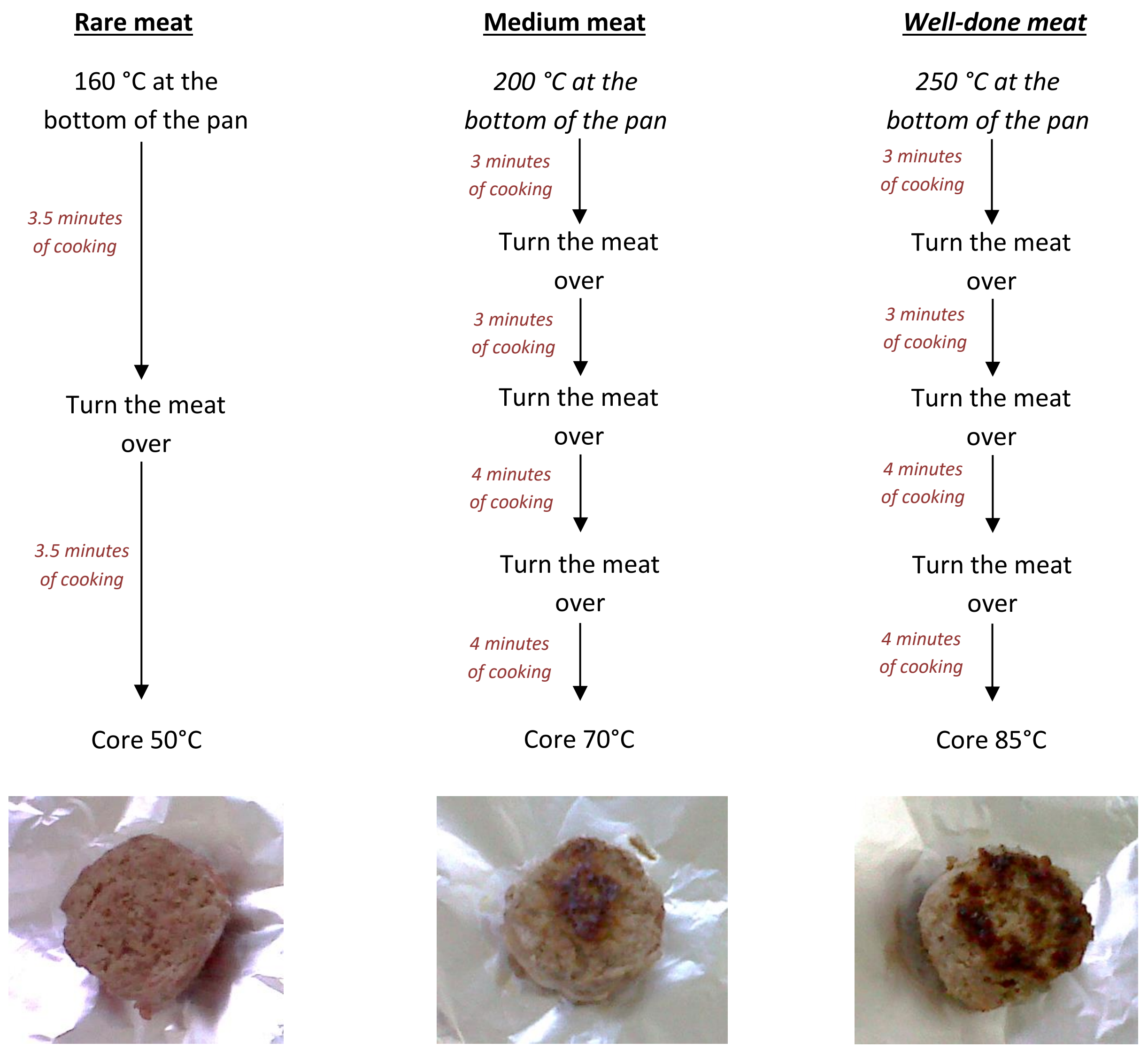

Figure S3. Cooking conditions used to simulate rare, medium or well-done meat.
